# What We Measure Matters: The Case of the Missing Development Data in Sendai Framework for Disaster Risk Reduction Monitoring

**DOI:** 10.1007/s13753-021-00382-2

**Published:** 2021-11-19

**Authors:** Ksenia Chmutina, Jason von Meding, Vicente Sandoval, Michael Boyland, Giuseppe Forino, Wesley Cheek, Darien Alexander Williams, Claudia Gonzalez-Muzzio, Isabella Tomassi, Holmes Páez, Victor Marchezini

**Affiliations:** 1 School of Architecture, Building and Civil Engineering, Loughborough University, Loughborough, LE11 3TU UK; 2 Florida Institute of Built Environment Resilience, University of Florida, Gainesville, FL 32661 USA; 3 Disaster Research Unit (DRU), Freie Universität Berlin, 12165 Berlin, Germany; 4 Stockholm Environment Institute Asia, Bangkok, 10330 Thailand; 5 School of Geography, University of Nottingham, Nottingham, NG7 2RD UK; 6 Institute of Disaster Mitigation for Urban Cultural Heritage, Ritsumeikan University, Kyoto, 603-8341 Japan; 7 Department of Urban Studies and Planning, Massachusetts Institute of Technology (MIT), Cambridge, MA 02142 USA; 8 Ámbito Consultores Limitada, 3565 202 Santiago, Chile; 9 Laboratoire Triangle Lyon, École Normale Supérieure de Lyon, 69342 Rhône-Alpes, France; 10 Department of Civil Engineering, Pontificia Universidad Javeriana, 110231 Bogota, Colombia; 11 National Centre for Monitoring and Warning of Natural Disasters (CEMADEN), São José dos Campos, SP 12247016 Brazil

**Keywords:** Development dynamics, Disaster risk root causes, Sendai framework for disaster risk reduction, Sustainable development goals

## Abstract

The Sendai Framework for Disaster Risk Reduction 2015−2030’s (SFDRR) framing moved away from disaster risk as a natural phenomenon to the examination of the inequality and injustice at the root of human vulnerability to hazards and disasters. Yet, its achievements have not seriously challenged the long-established capitalist systems of oppression that hinder the development leading to disaster risk creation. This article is an exploratory mapping exercise of and a collective reflection on Sustainable Development Goals (SDGs) and SFDRR indicators—and their use in measuring progress towards disaster risk reduction (DRR). We highlight that despite the rhetoric of vulnerability, the measurement of progress towards DRR remains event/hazard-centric. We argue that the measurement of disaster risk could be greatly enhanced by the integration of development data in future iterations of global DRR frameworks for action.

## Introduction

The main mechanism for promoting and implementing disaster risk reduction (DRR) globally is the Sendai Framework for Disaster Risk Reduction 2015–2030 (SFDRR). Agreed by the United Nations (UN) member states in 2015, the SFDRR replaced the Hyogo Framework for Action 2005−2015 (HFA), which, in turn, replaced the 1994 Yokohama Strategy and Plan of Action for a Safer World (de la Poterie and Baudoin 2015).

The SFDRR explicitly recognized what the previous frameworks failed to recognize—that is, that inequality and poverty are direct drivers of vulnerability to disasters, and thus disaster risk is inseparable from development dynamics (UNDRR 2015a). Therefore, the SFDRR is aspirational and focuses on the examination of the inequality and injustice at the root of human vulnerability. Achieving its aims requires collaborative and decentralized effort. But in the past six years, the long-established systems and ideological structures, within which we operate, and which hinder development—for example, neoliberalism,^1^ neocolonialism,^2^ patriarchy, globalization, racism—have not been seriously challenged or overturned. Instead, as we “build-in” and “re-build” development, more people than ever are affected by disasters, raising the question whether we are actually making progress towards DRR, and whether the SFDRR acts as an enabler of progress.

In this study we explored whether the underlying drivers of increased vulnerability, represented in the development data within the Sustainable Development Goals (SDGs), could be used for measuring how progress towards DRR is defined in the SFDRR. By undertaking a collective exploratory mapping exercise of indicators of the SDGs and the SFDRR, we discuss the disconnect between the two sets of indicators, which highlights that the United Nations Office for Disaster Risk Reduction’s (UNDRR) definition of disaster does not match the approach to measuring disaster risk. Although the rhetoric of the SFDRR shows an appreciation of the root causes of risk, the measurement of progress (the data collected) towards DRR remains event/hazard-centric rather than being rooted in a vulnerability and development (root cause/risk creation) approach. Moreover, we show that, while disaster risk data inform the SDGs, there is no mechanism by which development data inform the SFDRR. We argue that the measurement of disaster risk could be greatly enhanced by the integration of development data in future iterations of global DRR frameworks for action.

## Background

Before delving into the discussion around the indicators, the following sections set out why and how developmental agenda is important in the context of disaster risk. We first briefly trace the history of development in the twentieth century, then remind the reader about the definition of disasters, to finally demonstrate that development and disasters are intertwined—which is critical when indicators are created and the progress towards disaster risk reduction is measured.

### Development Agenda in International Policy

As Escobar (1999, p. 382) dissected, “development is a very real historical formation, albeit articulated around an artificial construct (underdevelopment) and upon a certain materiality (the conditions baptized as underdevelopment), which must be conceptualized in different ways if the power of the development discourse is to be challenged or displaced.” After the Second World War, the rise of international development has been a professed tool to even out the inequalities of a global capitalist economy. The way to do that has been through achieving prosperity—and the programs introduced by the World Bank and the International Monetary Fund (IMF) are indicative of this approach. But as the World Bank’s place on the international stage grew, its development role began to expand beyond what John Maynard Keynes had initially advocated and began to concern itself with equality, well-being, and social change (Prashad 2012; Mirowski 2013; Beddeleem 2020), remaining within the bounds of capitalism, and tied inextricably to economic growth, inequality, and poverty. But because the World Bank is tied directly to the centers of power in a global capitalist economy, it cannot act to redress the inequalities that fuel that economy, but rather is called upon to reproduce them (Taylor 2005). This may seem like a contradiction, and it is. This contradiction lies at the heart of the ideology of development.

The focus of international development was largely on poorer countries (that is, the Global South) and was grounded in the historical ties to particular parts of the Global South’s post-independence—such as the Commonwealth, Francophonie, or Iberoamerica. After the 1990s, there was a noticeable shift towards global development, as a result of increasing economic and trade integration and a rising concern over the systemic risks introduced by globalization. The aspiration of global development is to create a common future, recognizing the interdependence of different countries in producing public goods and confronting public ills (Currie-Adler 2014). This is reflected in the United Nations development agenda introduced by the Millennium Development Goals (MDGs) in 2000 and replaced in 2015 by the SDGs.

The concept of “sustainable development” has come to underpin broad policy goals in recent decades, culminating in the 2030 Agenda for Sustainable Development and the SDGs. As a concept, sustainable development is often credited to the 1987 Report of the World Commission on Environment and Development, *Our Common Future*, known as the Brundtland Report (WCED 1987). Prior to this, however, the 1972 UN Conference on the Human Environment sought to motivate countries to prioritize environmental issues, safeguard resources, and meet development goals in responsible ways. With limited progress and achievement on these fronts, the 1992 Earth Summit in Rio de Janeiro was held 20 years later, leading to the Rio Declaration on Environment and Development. This was followed by the 2012 Earth Summit, resulting in the “Future We Want” document that makes various recommendations, including the establishment of the SDGs. These global policy processes and frameworks have played important roles in shaping and framing “sustainable development.”

The Brundtland Report (WCED 1987, p. 15) defines sustainable development as “development that meets the needs of the present without compromising the ability of future generations to meet their own needs.” Crucially, “needs” is further explained as referring specifically to the “essential needs of the world’s poor, to which overriding priority should be given” (WCED 1987, p. 41). The principle of equitable access to resources for sustained growth and the role of participatory decision making in equity are also outlined and key to sustainable development strategies, which the report sought to be achieved by 2000.

But, of course, sustainable development was not and has not been achieved, despite the calls of this report and subsequent mechanisms such as the MDGs and SDGs. The MDGs lacked some fundamental dimensions, including environmental sustainability, equality, social inclusion, and governance (Peters et al. 2016). The SDGs therefore aim at continuing the unfinished work of the MDGs, better capturing the complexity of inclusive and sustainable development for all, and monitoring progress towards development by new measurements and a series of complex and interconnected indicators (UN 2020).

In this way, the SDGs set the benchmark by which the whole global development governance agenda is compared. The SDGs include 17 goals, 169 targets, and 246 (232 unique) indicators that cover a broad range of development issues that should be addressed by 2030. Different international organizations (Asia-Pacific Development Bank, Inter-American Development Bank, Organisation for Economic Co-operation and Development, UN, among others) have been promoting the creation of synergies among private and public actors and national and international institutions to provide countries with a strong support mechanism towards the implementation of SDGs and the achievement of their targets. A supposed successful implementation of the 2030 Agenda requires a balance across all areas of the social, environmental, and economic pillars of the SDGs, including socioeconomic progress and prosperity, the responsible use of the planet’s finite resources and fragile ecosystems, the response to climate change through adaptation and mitigation, as well as human security. These organizations and countries track the status of sustainable development efforts through a composite sustainability index and scorecards that are used also to compare the performance of countries. Notwithstanding these efforts, and as highlighted by Hickel (2019), Lamichhane et al. (2021), and others, the SDG agenda runs the risk of following the same route of the sustainable development discourse, as it prioritizes economic growth over social and political goals, and avoids challenging the status quo, norms, and the praxis of growth based on the exploitation of natural resources and human welfare. Moreover, there has been little progress in achieving the environmental dimensions of the SDGs as well as trade-offs between SDGs; and the COVID-19 pandemic has upset whatever progress existed even further (Hickel 2020b; Zeng at al. 2020).

### Definition of Disasters

The United Nations Office for Disaster Risk Reduction (UNDRR) defines disaster as “a serious disruption of the functioning of a community or a society at any scale due to *hazardous events interacting with conditions of exposure, vulnerability and capacity*, leading to one or more of the following: human, material, economic and environmental losses and impacts”' (UNGA 2017; authors’ emphasis). It is not just an occurrence of a hazard but “a disruption of the functioning of a society” because of what UNDRR calls “other risk factors” (see their annotation to the definition of “natural hazards”), meaning that if an earthquake happens in an uninhabited area, it is not typically considered a disaster. Yet, such a definition does not provide a robust explanation of the root causes that generate risk socially and politically (Oliver-Smith et al. 2017; Chmutina and von Meding 2019)—although this is somewhat covered in a separate definition for “underlying disaster risk drivers”—“Processes or conditions, often development-related, that influence the level of disaster risk by increasing levels of exposure and vulnerability or reducing capacity” (UNGA 2017).

It is also important to recognize that risk is a complex concept. While risk is traditionally defined as a combination of an impact and a likelihood, this definition must expand when we consider disaster risk as it cannot be examined without: analyzing historical sociopolitical trends; the political will to deal with risks; the capacity to map and assess the frequency of hazard events; the susceptibility to loss across a range of population groups and sectors; the capacity to take actions to prepare and to mitigate, to monitor the results of actions, to learn from successes and failures, and to maintain vigilance and foresight through more quiescent periods, when public interest will decrease (Wisner 2016). These elements are summed up in an equation-like mnemonic (Wisner et al. 2012):

$${\text{R }} \approx \, [{\text{H}}^{^{\prime}} \left( {{\text{V}}/{\text{C}}} \right) \, {-}{\text{ M}}]$$

where R stands for disaster risk, H for the specific hazard probability, V for vulnerability, C for localized and individual capacity for self-protection and recovery, while M “symbolises larger-scale risk mitigation by preventive action and social protection” (Wisner et al. 2012, p. 24)—M ideally complements and supplements C and should not act to block or dilute C; however, in reality this happens quite often! The elements are dynamic and are affected by global, national, and local economic and geopolitical change. They reflect long-established societal systems and ideologies. These elements define the context within and because of which disasters unfold. However, this is not how disaster risk is measured in reality.

The current DRR frameworks were initiated by the international community through the UN, have guided disaster related policies since 1995, and are intended to do so until the year 2030 (when a new framework will perhaps be introduced). This would indicate that representatives of most countries understand the complex nature of disaster risk and the web of social relations and vulnerabilities^3^ involved.

### Disasters and Development are Interconnected

Development ideas have been prominent in driving the formation of DRR discourse and practice, and gradually have become intertwined. Certain ideas were imposed on the fledgling DRR field: economic growth as a prerogative (Cuny 1994; Lewis 1999; Lewis and Kelman 2012), or the West acting as the “saviour of the world,” while erasing the wants and needs of those “underdeveloping” countries we still often refer to as developing (Galeano 1970; Chang 2009; Prashad 2012). These ideas are clearly reflected in the “Building Back Better” narrative, where the focus on “build back” is largely measured in economic terms (for example, investments and infrastructure) (Cheek and Chmutina 2021), by for instance prioritizing the speed of the reconstruction of buildings over the safety or livelihoods and neighborhoods (Kennedy et al. 2008; Zanotti 2010; Thomalla et al. 2018), and reproducing conditions of inequalities and vulnerabilities (Stone 1989) instead of alleviating those existing since prior to the disaster. Sometimes these processes are entirely predictable based on power differentials and the stated political goals of the institutions that set the terms of engagement (Stone 1989; Matthewman 2015).

The limits of GDP growth have been apparent for a long time (see, for instance, Piling 2018). Yet, at the international level, the development policies that are offered as solutions to disaster recovery are the same policies that were implemented prior to the disaster. Free-market systems influence pre-disaster contexts. For many governments as well as individuals the choice between increased exposure to disasters and relative safety is largely influenced by economic concerns. By arguing that disasters are “unsolved development problems” (Cardona 2004, p. 49), we fail to recognize that current terms of engagement allow the powerful to frame disasters as “a disruptor” of development, while disasters are a result of development under the current status quo.

Globalization and the “development of underdevelopment” (Frank 1966) have produced risk around the world. Vulnerability is a process as well as a result of globalized development. As Matthewman (2015, p. 136) observed, “Events are merely processes made visible.” Using this viewpoint, disasters taking place within the neoliberal paradigm are visual evidence of neoliberalism as a process. Within this dynamic lies a fundamental tension: disasters can be a threat to the state, yet neoliberalism drives the production of vulnerabilities. Neoliberalism exacerbates the tendency for governments to create risk in the pursuit of growing their economies. This risk creation can harm populations and have desultory effects on regions. The uneven development thus leads to a retrenchment of the problems that lead to the disaster in the first place.

Current approaches, however, see disasters as a one-off “event” rather than as a sociopolitical process (Fuentealba 2021). Such a hazard-centric approach is reflected in DRR measures that can displace the disaster temporarily but leave the underlying factors that created the risk that leads to the disaster unaddressed. What is accomplished is neoliberal restructuring—privatizing public assets, deregulating major industries, reducing corporate taxes, competition of capital investments, the conflation of democracy with free-markets, scepticism of government planning and policy making that addresses social inequality, elimination of the concept of the public good—which is traded in for a belief in personal responsibility, a belief that the government’s job is to assist the private sector (Cheek and Chmutina 2021). The nature and degree of these factors varies by state and disaster, but the fundamental elements are there. These fundamental elements must be considered if DRR is to address the developmental challenges.

## Methodology

The SDGs reflect developmental challenges that contribute to root causes of disasters. This article locates the problematic nature of these approaches within the SFDRR. In the time since the framework’s implementation in 2015, duty-bearers around the world have worked to compile data according to the metrics devised, with the goal of reducing disaster risk. But disaster risk cannot be addressed without looking at root causes, and the rhetoric of the SFDRR does not filter through to the way it measures progress towards DRR, retaining an event- and hazard-centric approach to risk.

To understand what underlying drivers of disaster vulnerability are represented in the development data within the SDGs, and whether such essential data for measuring progress towards the reduction of disaster risk are reflected in the SFDRR, the authors collectively explored each of the 246 SDG^4^ indicators (grouped in 17 goals, 169 targets) and 38 SFDRR indicators to scrutinize the relationship between all SFDRR and SDG indicators. It was not our aim to provide a “scientific” analysis of the relationship. We wanted to explore, through extensive discussions, the extent to which each SDG indicator is connected to or addresses root causes of vulnerability and is grounded in an axiom that disasters are not natural (Kelman 2019). We adopted the widely used Pressure and Release (PAR) model, proposed initially by Blaikie et al. (1994) and refined later by Wisner et al. (2004, 2012), to frame our discussions and guide our thinking through the structural factors—that is, economic, political, cultural—in society such as “social relations” and “structures of domination” as constitutive elements of disasters and risks (Wisner et al. 2004). From this perspective, root causes of disaster vulnerability were framed as:

[A]n interrelated set of widespread and general processes within a society and the world economy. They are ‘distant’ in one, two or all of the following senses: spatially distant (arising in a distant centre of economic or political power), temporally distant (in past history), and finally, distant in the sense of being so profoundly bound up with cultural assumptions, ideology, beliefs and social relations in the actual lived existence of the people concerned that they are ‘invisible’ and ‘taken for granted’ (Wisner et al. 2004, p. 52).

We used this framing to map out the potential of SDG indicators to provide data on underlying causes of disaster vulnerability.

In the summer and autumn of 2020, 20 researchers and practitioners from different countries, all experts in disaster studies (but with different academic backgrounds in construction, urban planning, sociology, architecture, politics, human geography) got together biweekly to explore which SDG indicators are explicitly linked to disaster vulnerability and should thus be incorporated into the SFDRR indicators to inform effective DRR progress. The exploration was through a discussion, going through goals and targets and finding consensus about the relationships between SDGs and disaster risks. As a result of these discussions, the indicators were divided into four groups:

1. SDG indicators that are explicitly connected to the SFDRR: these largely focus on loss and are already shared between the SDGs and the SFDRR (for example, SDG 1.5.1 Number of deaths, missing persons and directly affected persons attributed to disasters per 100,000 population);
2. SDG indicators that can significantly inform on underlying causes of disaster vulnerability: these would commonly be understood as vulnerability in a way that is defined by the UNDRR^5^ (for example, SDG 1.4.1 Proportion of population living in households with access to basic services);
3. SDG indicators that have some connection to underlying causes of disaster vulnerability but may not be explicitly considered as such (for example, SDG 2.2.2 Prevalence of malnutrition among children under 5 years of age, by type); and
4. No explicit or significant connection between the SDGs and the SFDRR.

Once the links were established, we were able to see the disconnect between the definition of a disaster (that focuses on vulnerability and its underlying root causes) and the way that progress toward disaster risk reduction is measured (which is hazard-focused and is mostly about impact and loss data). The core themes of this disconnect are presented in the following section.

## Connecting the Sendai Framework for Disaster Risk Reduction 2015−2030 (SFDRR) and the Sustainable Development Goals (SDGs)

The 2030 Agenda has led to the formal adoption of SFDRR indicators by the SDG process, with many people viewing the former as a “how to” guide on implementing the higher-level disaster-related SDG goals (Peters et al. 2016). The UNDRR (2019) states that, “Both the SFDRR and the SDGs outcomes are a product of interconnected social and economic processes. As such, there is a lot of synergy between the two policy instruments.” More specifically, according to the UNDRR (2015b), “there are 25 targets related to DRR in 10 of the 17 SDGs.” Three SDGs (Goals 1, 11, and 13, and their targets 1.5, 11.5, 11.b, and 13.1) have been adopted by the SFDRR (UNDRR 2015a), thus for the first time explicitly connecting some of the objectives of both international instruments. These SDG targets are directly relevant to five SFDRR targets (A to E): A (small) number of SDG and SFDRR indicators are shared; these include indicators 1.5.1 to 1.5.4, 11.5.1 and 11.5.2, 11.b.1 and 11.b.2, and 13.1.1. However, this does not happen beyond the indicators that measure disaster losses or explicit implementation of DRR strategies at a national level.

Clearly, SDG indicators can play a similar role in informing how we measure progress towards disaster risk reduction. Health (SDG 3), gender disparities (SDG 5), poverty (SDG 1), denied access to education (SDG 4), land tenure (SDGs 1 and 11), access to services (SDGs 6, 7, and 11), among others, are all manifestations of root causes of disaster risk (Cannon 2008; Bradshaw and Fordham 2013; Wisner 2016; Sarmiento et al. 2020) and could inform or address root causes of disaster risk. Yet, there is no incorporation of development data by the SFDRR, despite its rhetoric.

Our exploration reveals that the relationship between the SFDRR and the SDGs is dominated by an approach, where quantitative monitoring of DRR progress—beyond the conceptual discussion on disaster-development interplay—is not considering available development indicators, represented most prominently in the SDGs (which could have been done, given that the SDGs were implemented 15 years before the SFDRR). The SFDRR’s guiding principles reflect the idea of development (UNDRR 2015a, p. 13):

- Guiding principle C: “Managing the risk of disasters is aimed at protecting persons and their property, health, livelihoods and productive assets, as well as cultural and environmental assets, while promoting and protecting all human rights, including *the right to development*” (authors’ emphasis);
- Guiding principle H: “Disaster risk reduction is essential to achieve *sustainable development*” (authors’ emphasis);
- Guiding principle J “Addressing underlying disaster risk factors […] contributes to *sustainable development*” (authors’ emphasis).

In fact, the SFDRR uses the word “development” 103 times on its 27 pages; but development data are not then utilized in SFDRR progress measurement. Development is interpreted in the SFDRR as it is interpreted in other international frameworks—it is rooted in growth of GDP (Stiglitz et al. 2009; Costanza et al. 2014), regardless of the fact that the assumption that global growth will facilitate the achievement of key social goals—such as reducing poverty and hunger, and increasing education—is incorrect as it ignores distributional concerns and only weakly correlates with well-being (Hickel 2018; Pilling 2018). The SFDRR thus fails to meaningfully deal with the root causes of risk, which becomes clear when we unpack the common threads of disaster risk creation as our exploration shows.

Take, for example, the case of informal settlements in Chile and the SDG 11.1.1 Proportion of urban population living in slums, informal settlements or inadequate housing. Informal settlements represent vulnerability, which is generally expressed in various ways of precariousness—physical, spatial, social, and economic (Sarmiento et al. 2019). In Chile, between 2011 and 2018, it was estimated that the number of families living in informal settlements or *campamentos* grew by 57.1%, from approximately 27,000 to 43,000 households in seven years (TECHO 2018). Another cadastre by the Ministry of Housing and Urban Development (MINVU) detected 802 informal settlements where 112,000 people live (MINVU 2020). According to Sebastian Bowen (24 Horas TVN Chile 2021), Executive Director of TECHO, these figures are already “obsolete” as the Covid-19 pandemic increased the number of households living in precarious settlements to over 63,000 during 2020. In July 2020, the Ministry of National Assets (MBN 2020) reported an increase of 2.1% in the amount of land illegally occupied—299 more plots than in December 2019. Among the informal settlements registered by MINVU as of 2019, 60 were in areas directly exposed to fire, landslides, or flooding (MINVU 2020). The SDG 11.1.1 could thus provide an important insight for the SFDRR progress in terms of disaster risk reduction and creation.^6^

This reveals the shortcomings of the SFDRR, not just in providing a more comprehensive picture of disaster risk today, but also in missing the opportunity to have a longer view on how risk is produced throughout space and time. The SFDRR indicators shift attention away from systemic injustice, inequality, unsustainability, and exploitation, thus neglecting the economic and political conditions that produce vulnerability (Pulhin et al. 2021). The SFDRR does not enable practitioners to identify and grapple with processes that produce vulnerability, that is, the underlying root causes that are not addressed, as our exploration shows*.* The SFDRR has been operationalized with the hope of being a part of “progress” toward less disaster impact. It promotes consideration of the root causes through its language and points to its interrelation with development. Tracing the progress towards many SDGs would help to show how disasters are created over time. Instead, the shared indicators only focus on impact/losses (in monetary values or numbers of casualties), disregarding the concepts of vulnerability and capacities (that nevertheless are highlighted in the definition of a disaster used in the SFDRR).

The problem with claims that fewer people are affected by disasters is that the overall framing of such reporting is hazard- and event-focused. Despite scientific data to the contrary, the popular narratives of disasters still portray them as a “sudden” “unexpected” “shock,” instead of a sociopolitical process. Therefore, a country might report less injury, property damage, and livelihood loss from disaster “events”—and yet be creating everyday risk and precarity for its citizens. People might lose access to life’s essentials and be at a much higher risk of being “affected”—but we would never realize it until it was too late, and a large earthquake, flood, or storm discriminately impacted them. The SFDRR’s dominant approach is to measure the impacts of “disaster events,” and this is where we believe that, as a global community, we can do much better to create tools that would actually help reducing disaster risks.

Since our present collective understanding of disaster is increasingly decentering the primacy of the hazard, and instead centering the role of populations experiencing “vulnerability,” we must be consistent, and therefore follow this vulnerability thread to the precarity people experience daily. If a community is grappling with precarious informal settlements, famine, gendered violence, income disparity, and corruption due to displacement, conflict, and loss following neoliberal policy shifts, such risk also impacts its capacities to deal with hazards. If we are to be ideologically consistent, we must follow this thread even as we make recommendations or implement interventions. An analysis that treats disaster risk as a distinct category of risk separate from daily structural dispossession and violence is a failed one. Furthermore, it is important to consider how risk and its components evolve over time and are generated by a complex series of root causes—often originating centuries ago (Blaikie et al. 1994; Oliver-Smith and Hoffman 2020; Rivera 2020)—mostly converging on those living in the most precarious situations.

## Discussion

What different SFDRR indicators demonstrate is that, while there has been a shift from hazard to vulnerability, the latter is still treated as an outcome that can be addressed through “building resilience” (for example, Cheek and Chmutina 2021) and not as a process that is influenced by external socioeconomic factors and is historically rooted. We know that the creation of risk is a complex process influenced by internal and external socioeconomic factors, local and global socioecological policies, and is geographically located and historically rooted—all of which are a manifestation of power. By failing to locate the question of vulnerability in a critique of power, the SFDRR positions itself as an “a-political” project. The SFDRR itself is not a public policy instrument. If we consider “politics” as “space and power,” as action in this same public space (Arendt 1958), these become the place of memory, discourse, and preservation of artifacts and knowledge (Tomassi 2017). In this sense, reducing the action of policies to their measurable impacts is a fundamental error that distorts the objectives and actions of the SFDRR.

Yet the impact of the SFDRR on public policies is significant and, therefore, much more intrusive than the interests of different powers in the field at different scales (local, national, international). In the global context of disaster risk, the SFDRR dominates the face of charity and development and acts not only at the scale of national governments but also in the relationship that national governments have with international bodies. This is an environment where interested actors lobby to maintain the economic status quo, that is capitalism. Without an analysis of the entangled priorities and underlying structures that drive the disaster risk reduction that the SFDRR aims to achieve, steer, and coordinate, its impact can only remain partial and superficial.

The SFDRR reproduces the same narrative scheme created for development from the post-war years, and unifies it with that of contemporary development. This narrative becomes an ideological tool as it contains the claim that progress is based on scientific truths, such as a-historical objects, and is to be re-dimensioned and contextualized as Canguilhem does in his 1969 lecture (Canguilhem 1969) when he distinguished scientific ideology from the ideology of the scientist. Marx (1843) retained, in the sense he gave to the term ideology, the concept of a reversal of the relationship between knowledge and the thing. However, in reality, SFDRR does not fully consider what development is—and how the already available data can be used for the development to be reflected in the progress towards DRR.

We see this clearly in DRR projects. The technical and “expert” way of conceiving and dealing with disasters makes some groups of people legitimate and convincing—while reinforcing the marginalization of other groups. Following Althusser (1971), we see how the SFDRR, built on a rigorous Western “scientific” approach to society and the problems it faces because of disasters, “represents” the “imaginary relationship of individuals” with the real world. This imaginary relationship is guided by a neoliberal narrative that normalizes vulnerability as “individual guilt” (von Meding 2021) and fetishizes the status quo of the social system, to which only few want to return (Cheek and Chmutina 2021).

## Conclusion

In this article we explored the extent to which the underlying drivers of increased vulnerability are represented in the development data within the SDGs, and whether such essential data for measuring progress towards the reduction of disaster risk are reflected in the SFDRR. We demonstrated that the current SFDRR approach to measuring DRR is event/hazard-centric—instead of locating the DRR process within broader development, as the rhetoric of the SFDRR makes clear should be the case. Despite the SFDRR’s claims of focus on development, what the framework actually measures is the impact of “disaster events.” Thus, it avoids dealing with the complexity of social, political, and economic interests that lead local, national, and international actors to grapple with each other over change, and therefore influence the notions of development as well as the realization of these notions.

So, is the SFDRR tool fit for its intended purpose? The SFDRR objective is to measure global progress in reducing disaster risk—but we contend that the choice to measure event impacts, rather than integrate data that would interrogate root causes of disaster risk, undermines the effectiveness of the framework. It still promotes the language of development that, as Amy Allen (2017) pithily noted, is the language of oppression and domination for two-thirds of the world population.

The effectiveness of the SFDRR has been questioned previously (Briceño 2015; Manyena 2016; van Niekerk et al. 2020; Wisner 2020), highlighting the limited impact and benefits provided by its implementation in different countries. Although civil society and experts have been calling for decentralization of DRR efforts, it remains rhetorical under the SFDRR. Instead DRR is largely controlled rigidly by upward accountability (or, in some cases, no accountability at all) to the central state or, alternatively, to a donor country or international nongovernmental organization (Ainuddin et al. 2013; Grady et al. 2016; Rumbach 2016). And as the pressures of multi-level governments to invest in mining, large scale agribusiness, smart cities and luxury housing development is increasing (Wisner 2020), the realization of “commitment to address disaster risk reduction and the building of resilience to disasters with a renewed sense of urgency within the context of sustainable development and poverty eradication” (UNDRR 2015a, p. 9) is becoming more and more nebulous. After six years, the implementation of the SFDRR does not show significant increased political will to weed out investments that are blind to risk from those that are risk-informed, despite the rhetoric of the SFDRR itself around root causes of vulnerability such as weak governance arrangements and non-risk-informed development. This accommodates the ideology and development model of neoliberalism and provides a foundation for disaster risk creation.

We are certainly not arguing that the SDGs are more effective than the SFDRR. We recognize the strong critiques posed in relation to the SDGs’ indicators and approach in terms of post-political representation, lack of reliable data at multiple levels of inquiry, and decontextualization of data (Sultana 2018; Hickel 2019, 2020a). Rather, we want to highlight that this missed opportunity for convergence should lead us to reflect on the usefulness of frameworks and on the validity of their claims to progress. We want to challenge disaster risk and development scholars, policymakers, and practitioners to reflect on the need to question the validity of large datasets that do not consider the structure of society as a baseline for decision making about disaster risk. These datasets can be useful, but only when they try to move a critique to the structural foundations of our society, something that has always been present in disaster scholarship.

To paraphrase Judith Butler (2016, 2020), here lies the opportunity to correct the error of essentializing precarious material conditions with particular identities. Instead, we must turn our gaze toward the mechanisms of oppression. We know that structural housing stability and access to safe utilities such as water and electricity are important when experiencing hazard events. If the population living in informal urban settlements is increasing, then risk along precarity lines can be said to be increasing. We know that gendered violence is connected to community experiences of disaster (Yoshihama et al. 2019). If femicide is increasing, then risk along gendered lines can be said to be increasing. We also know that income inequality is relevant in assessing livelihood precarity and hazard risk. If the aim of DRR is to reduce the number of people directly affected without addressing root causes, we are playing a dangerous technocratic game that can easily be thrown off by new hazards.

As the international community moves forward from the SFDRR, critical self-reflection should be ongoing. If the intention is truly to develop mechanisms to measure progress or setbacks on reducing risk, the need to move away from measuring “event” impacts is essential. We believe that the international community can live up to the rhetoric of the SFDRR, but any future framework must develop a process for integrating development data that are related to vulnerability and precarity as its central focus. This aligns with wider efforts in scholarship and practice to move away from an event- and hazard-centric framing of disasters to one that recognizes that disasters are time-delayed manifestations of structural violence and maldevelopment.

## Footnotes

1 While neoliberalism “carries no settled definitions” (Fraser 2019, p. 17), it is perhaps best described by Peck (2010, p. xii) as “an open-ended and contradictory process of politically assisted market rule…. [It] is not the antithesis of regulation, it is a self-contradictory form of regulation-in-denial”.

2 Similarly, there is no singular definition of neocolonialism, yet its characteristics are perhaps best summarized by Kwame Nkrumah that this concept warns us of the potential regressive impact of unregulated forms of aid, trade, and foreign direct investment in relation to poverty reduction and well-being in the Global South (Nkrumah 1965).

3 In this article we in particular focus on vulnerability as a lens to highlight root causes that turn hazards into disasters, and that are grounded in developmentalism. It is however beyond the scope of this article to critique vulnerability as a concept.

4 The analysis of SDG indicators was carried out based on the Tier Classification for Global SDG Indicators as of 17 July 2020 (updated later on 26 October 2020 to include custodian agencies missing from the table). The original list of all SDGs and their indicators is available at: https://sdgs.un.org/goals.

5 The UNDRR Terminology Glossary (UNDRR 2017) defined vulnerability as “the characteristics determined by physical, social, economic and environmental factors or processes which increase the susceptibility to the impacts of hazards.”

6 This indicator however overlooks the root causes that force people to move into informal settlements in the first place (for example, unequal access to resources and opportunities)but this could be informed by other SDG indicators.
